# Linear and Branched PEIs (Polyethylenimines) and Their Property Space

**DOI:** 10.3390/ijms17040555

**Published:** 2016-04-13

**Authors:** Claudiu N. Lungu, Mircea V. Diudea, Mihai V. Putz, Ireneusz P. Grudziński

**Affiliations:** 1 Department of Chemistry, Faculty of Chemistry and Chemical Engineering, Babes-Bolyai University, 400028 Cluj, Romania; lunguclaudiu5555@gmail.com (C.N.L.); diudea@gmail.com (M.V.D.); 2 Laboratory of Structural and Computational Physical-Chemistry for Nanosciences and QSAR, Biology-Chemistry Department, Faculty of Chemistry, Biology, Geography, West University of Timisoara, Str. Pestalozzi No. 16, 300115 Timisoara, Romania; 3 Laboratory of Renewable Energies-Photovoltaics, R&D National Institute for Electrochemistry and Condensed Matter, Dr. A. Paunescu Podeanu Str. No. 144, RO-300569 Timisoara, Romania; 4 Faculty of Pharmacy, Medical University of Warsaw, 02-097 Warsaw, Poland; ireneusz.grudzinski@wum.edu.pl

**Keywords:** chemical property space, QSAR/QSPR, linear PEI (LPEI), branched PEI (BPEI), molecular principal moment of inertia, geometric descriptors, topological descriptors

## Abstract

A chemical property space defines the adaptability of a molecule to changing conditions and its interaction with other molecular systems determining a pharmacological response. Within a congeneric molecular series (compounds with the same derivatization algorithm and thus the same brute formula) the chemical properties vary in a monotonic manner, *i.e.*, congeneric compounds share the same chemical property space. The chemical property space is a key component in molecular design, where some building blocks are functionalized, *i.e.*, derivatized, and eventually self-assembled in more complex systems, such as enzyme-ligand systems, of which (physico-chemical) properties/bioactivity may be predicted by QSPR/QSAR (quantitative structure-property/activity relationship) studies. The system structure is determined by the binding type (temporal/permanent; electrostatic/covalent) and is reflected in its local electronic (and/or magnetic) properties. Such nano-systems play the role of molecular devices, important in nano-medicine. In the present article, the behavior of polyethylenimine (PEI) macromolecules (linear LPEI and branched BPEI, respectively) with respect to the glucose oxidase enzyme GOx is described in terms of their (interacting) energy, geometry and topology, in an attempt to find the best shape and size of PEIs to be useful for a chosen (nanochemistry) purpose.

## 1. Introduction

The concept of chemical space, by its currently accepted definition, is the space spanned by all possible molecules/chemical compounds, with any stoichiometry of electrons and atomic nuclei, and any possible topology [1]. The topology of the chemical space is not unique, so a variety of isomers exhibiting similar/dissimilar properties may appear. Drug design and molecular design in general involve the screening of the chemical space [2] within which one may move by chemical reactions. A chemical property space is then a multi-dimensional space, where physico-chemical properties of a molecule (or a molecular system) have the highest probability of being found. In drug design, this property space coincides with the drug likeness [3]. Visualization/exploration of the chemical property space may be realized by theoretical (statistics like “principal component analysis” PCA, similarity maps, U matrix, clustering, diverse regression algorithms, generative topographic mapping GTM, radar plots, *etc*.) [4], while “moving” within this space may be accomplished by chemical reactions, resulting in functionalized/derivatized new molecules (or molecular systems). Each of these methods for exploring and/or moving within the chemical property space is applied specifically, according to the type of compounds under study [4]. In the present study, PEIs (*i.e.*, polyethylenimines) are computationally explored by their chemical property space; for this class of compounds, two-dimensional plots and radar plots are suitable for the design of receptor relevant subspace and for diversity analysis, respectively [4,5,6]. Using statistical methods in exploring the chemical property space of PEIs, their physico-chemical properties can be predicted even consecutively to derivatization and functionalization. In other words, the physico-chemical behavior of a class of compounds, when moved within its chemical space, can be predicted by computational methods [7]. To properly predict the behavior of the derivatized compounds, the properties of the parent compounds must be computed [8].

PEIs (polyethylenimines) are polymeric molecules composed of repeating units of amine groups and two aliphatic carbons; a branched PEI, BPEI, may have all types of primary, secondary and tertiary amino groups while a linear PEI, LPEI, contains only secondary and primary amino groups. LPEI is solid at room temperature (melting point about 73–75 °C) while BPEI is liquid, irrespective of the molecular weight [9]. LPEI is soluble in hot water at low pH, and also in chloroform, ethanol or methanol. PEI has many applications, due to its polycationic character. The PEI property as a transfection reagent is well-studied; polyethylenimine was the second polymeric transfection agent discovered (after poly-l-lysine) and it binds to anionic residues of DNA by its positively charged units [10]. PEI is, however, cytotoxic [11].

## 2. Computational Methods

In order to study PEIs, their chemical property space, *i.e.*, the properties variation consecutive to functionalization and derivatization, were developed to simulate the interaction of PEIs with molecular systems (*i.e.*, nano-devices) for a set of PEI molecules. The method used in designing the PEIs series was (structural) derivatization (e.g., C–C–N unit repetition), producing branched (B) and linear (L) PEIs isomers. Two congeneric PEIs series, namely C14N8 and C18N10 have been designed; each group consists of 4 members, one LPEI and three BPEIs, LPEI 01 and BPEI 02 to 04 (with different branching). These molecules were studied both as free entities and parts of a nano-system (*i.e.*, derivatized/functionalized). The degree of functionalization/derivatization of PEIs is reflected in its chemical property space by a property variation. As for the degree of functionalization used in modeling these nano-therapeutic systems, several “generations” (*i.e.*, gen. I, II, III, *etc*.) may be distinguished within the complex molecular architecture. In the congeneric series of PEI (C14/C18), the magnitude of properties variation enabled the differentiation between the two PEI series.

The properties by which variation is relevant in our case reflect variation in the molecular structure: atomic distance, angles, and dihedral angles, with consequences in the energetics (reactivity *vs*. stability) of the studied molecular systems. In this respect, for each group member (of PEIs), a docking study was performed, using GOx (glucose oxidase) as a receptor. A crystallographic structure of GOx (PDB ID 3QVR) imported from the protein data bank [12], with a resolution of 1.3 Å, was used. Energy minimization and partial charges were performed using an MMFFF94 force field integrated in an online server [13,14]. The site where docking was performed was the active site of GOx for glucose oxidation [15]. All GOx valences, floods, charges, were corrected and the water molecules, other ligands and cofactors were removed. Docking of PEIs to GOx was performed using AutoDock software [16].

Dihedral angle was chosen as the monitored property, since dihedral angle values are sensitive to changes in molecule geometry subsequent to molecular interactions and because they correlate with torsion angles and the system free energy [17].

**Figure 1:**
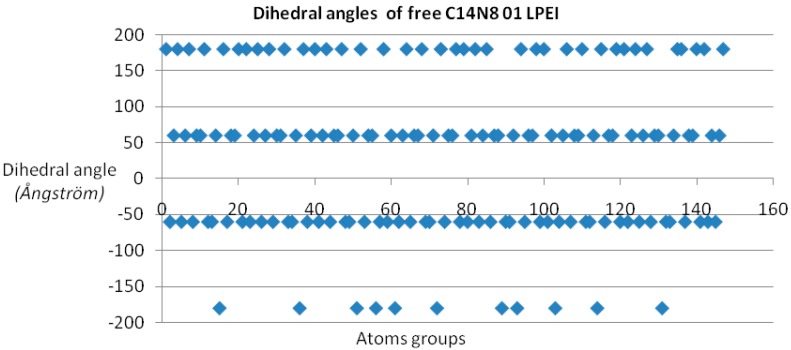
Dihedral angles of free linear polyethylenimine (LPEI) 01 C14N8, represented as a function of the atom groups; more details in the main text.

**Figure 2:**
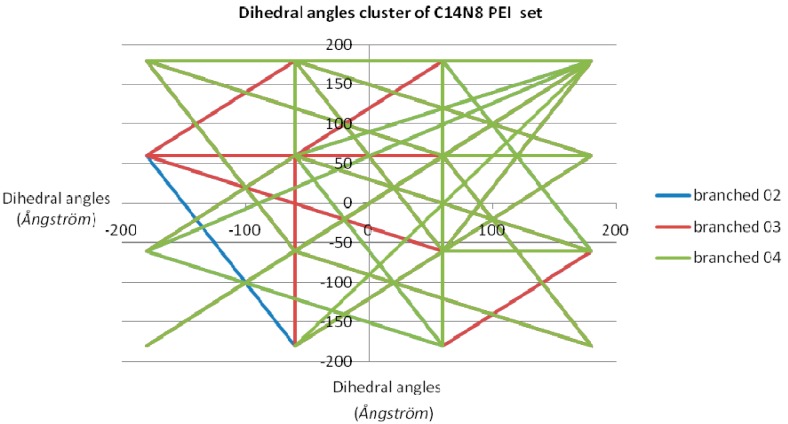
Dissimilarity of LPEI 01 *vs*. BPEIs in the C14N8 set (see additional material for the rest of the clusters obtained for C18N10 and C14N8 & C18N10).

Dihedral angles [17] were calculated for each member of the two PEI groups, both in the free and docked ligand molecules, and represented as scatter plots (see Figure 1). In order to have a measure (and an illustration) of variation within the chemical property space, the “pairs of values comparison” method [18] was used (by Office 2007 software package) on the dihedral angle values of each LPEI/BPEI pair [19,20]; the resulting “dissimilarity cluster” representation is shown in Figure 2. The dihedral angle values of L 01-C14N8 PEI were chosen as the benchmarking term; next, pairs of values were compared, with terms in C14N8 series, in C18N10 series and in the combined (C14N8 &C18N10) series. More dissimilar pair molecules provide more connections in the cluster graph representation, with a distinct color for each analyzed pair [17,21].

**Figure 3:**
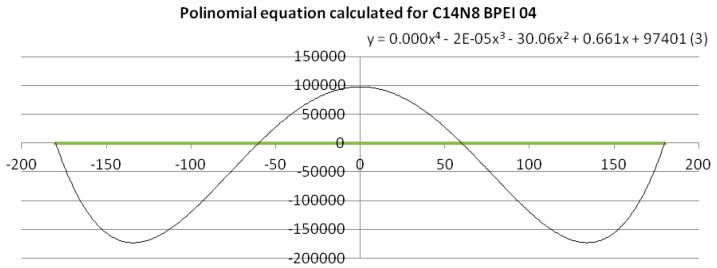
Polynomial equation calculated for the dihedral angles cluster of C14N8 BPEI 04 (the “(3)” number of equation corresponds with that of the Supplementary Material).

To get a quantitative measure (mathematically expressed) of the dissimilarity of PEIs dihedral angles, a fourth-degree equation based on a polynomial trend line with period 4 was calculated [22]. An example of polynomial trend line and data linearization is given in Figure 3 (see also the Supplementary Material).

To represent the chemical property space (a multivariate one), two methods were chosen: (i) a 2D surface shape and (ii) a radial chart. In this respect, ten quantitative variables (including topological and geometric descriptors) were computed and represented as discussed above.

**Figure 4:**
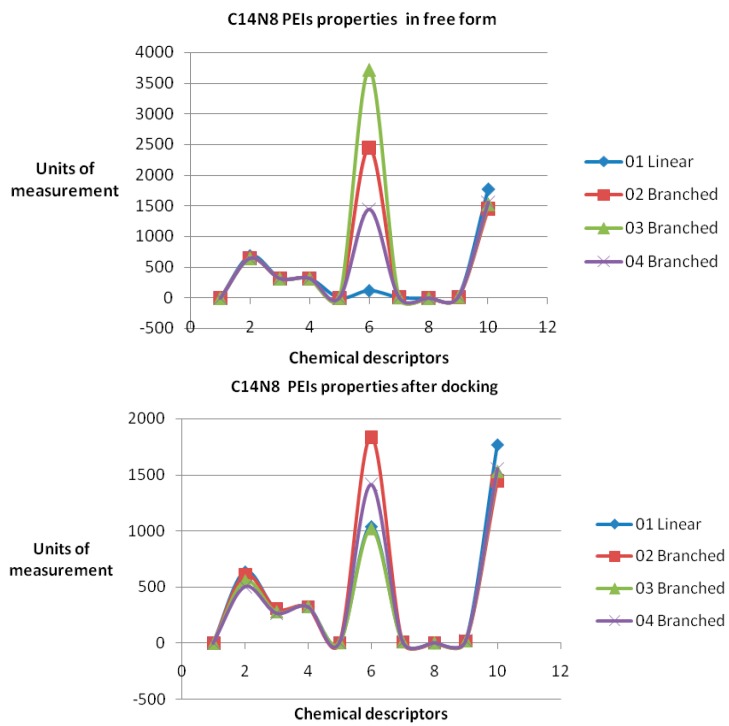
Chemical properties variation for C14N8 L/B PEIs **i**n the free (undocked)—(**top**), and in docked form—(**bottom**). 1—log *P*; 2—Connolly accessible area (Å^2^); 3—Connolly molecular area (Å^2^); 4—Molecular weight; 5—Ovality; 6—Principal moment of inertia; 7—Molar refractivity (cm^3^/mol); 8—Partition coefficient; 9—Topological diameter (bonds); and 10—Wiener index.

**Figure 5:**
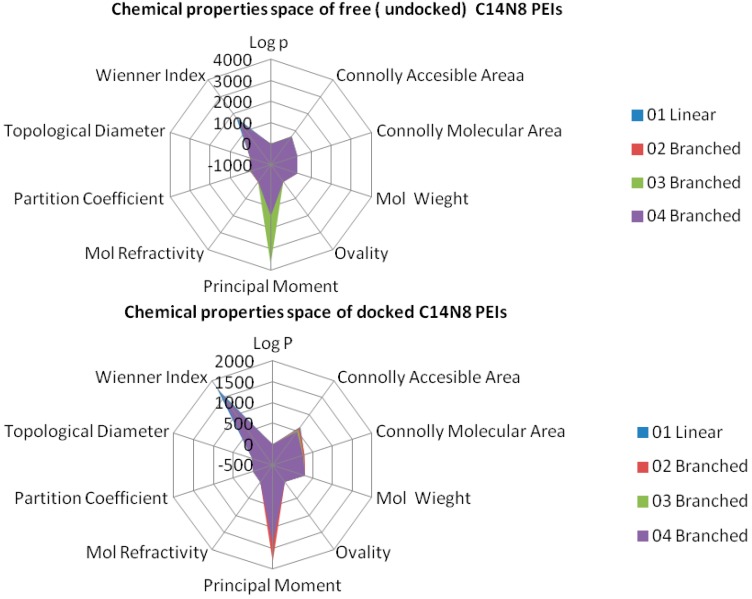
Chemical property space of C14N8 PEIs before (**top**) and after docking (**bottom**).

Data, computed for both docked/undocked PEIs, include the following variables: Connolly molecular area, Connolly accessible area [23], molecular weight, ovality, inertial principal moment, molecular refractivity, topological diameter and Wiener index, log *P* and another partition coefficient [24,25]. Examples of chemical property variation and chemical property space of PEIs group C14N8 are shown in Figure 4 and Figure 5 (see the Supplementary Material for the rest of the series).

In order to determine the maximum length of the chain of PEIs docked on GOx, an additional docking study was performed. A set of LPEI molecules, from C2 to C70 (see Supplementary Material), were docked at the enzyme catalytic active site (see the above described docking method). After docking, QSAR models were developed, with the steric energy of the GOx-PEI complex being the dependent variable. Independent variables were the number of C atoms, number of N atoms, number of hydrogen donor groups (HD), the mean distance between two hydrogen donor groups (HD-HD-Mean), the maximum distance between two hydrogen donor groups (HD-HD-Max), and Wiener index. A multiple linear regression (MLR) method was used. The model was validated using the leave-one-out method.

Total hydrogen bonds energy of the GOx-PEI complex together with the steric energy of PEI and torsions of PEI after docking were also calculated in order to give a more detailed view of the GOx-PEI interaction. Data were collected for the C14N8 and C18N10 sets while the docking conditions were kept as in the previous studies.

## 3. Results and Discussion

Dihedral angle values show four distinct domains for all PEI undocked molecules (see Figure 1); in the case of docked PEIs, the dihedral angle values are distributed in a rather disordered picture, probably taking into account the PEI molecule “accommodation” according to the binding site shape [26].

Clusters obtained from “comparison of pair values” method applied on dihedral angle values for the studied PEIs showed a quite ordered distribution. Let us look at the graph in Figure 2, representing the dissimilarity cluster obtained for the C14N8 PEI set, with the values of LPEI 01 C14N8 dihedral angles, as the reference: the graph with respect to BPEI 02 has only one edge representing its dissimilarity to PEI 01; next, BPEI 03 C14N8 has 8 straight lines, meaning it is more distinct [27] from LPEI 01 in comparison to BPEI 02 C14N8; finally, PEI 04 C14N8 (green lines) has even more lines in the cluster of dihedral angles, making it the most dissimilar to the reference LPEI 01 C14N8. LPEI 01 itself is not represented on the chart, being the comparison (benchmarking) term.

To give a quantitative measure of the similarity/dissimilarity, qualitatively shown by the graph in Figure 2, a mathematical approach was used: a polynomial trend line of order 4 was calculated for the clusters belonging to BPEI 04 (C14N8 and C18N10), thus obtaining polynomials of the fourth degree (a total of 15 equations were calculated—see the Supplementary Material). The equations (see the Supplementary Material) are:

C14N8 BPEI 04: *y* = 0.0001*x*^4^ − 2 ° 10^–5^*x*^3^ − 30.06*x*^2^ + 0.661*x* + 97,401 (1)

C18N10 BPEI 04: *y* = 0.0001*x*^4^ − 1 ° 10^–5^*x*^3^ − 14.96*x*^2^ + 0.622*x* + 48,499 (2)

To exemplify such a function expressed by the fourth-degree equation, Equation (1), (3.C14) in the Supplementary Material, is represented in Figure 3; the polynomial trend line was calculated for the points belonging to BPEI 04 C14N8. When applied the (dis)similarity operation listed above, a line was obtained composed of all the points that represent the values of BPEIs 04 C14N8 dihedral angles that differ from those of the reference LPEI 01C14N8 (the green line). The polynomial equation for this line is a sinusoidal with 4 points of intersection with the line (see Figure 3), according to the degree of the equation. Such representations have been performed for all the cluster series (11 equations and 11 graphs, respectively—see the Supplementary Material).

Next, the roots of the above equations were calculated; they consist of real and imaginary numbers (see Table 1, for the equation shown in Figure 3). The roots calculus was done by using the online computational engine Wolfram Alpha [28].

To escape the complex roots we have eliminated the fourth and third degree terms of the equation, based on their negligible coefficients; thus, the equations (1) and (2) now become

C14N8 BPEI 04: *y* ≅ −30.06*x*^2^ + 0.661*x* + 97,401 (3)

C18N10 BPEI 04: *y* ≅ −14.96*x*^2^ + 0.622*x* + 48,499 (4)

For these new equations, the calculated roots were all real numbers (Table 2).

The obtained roots fall in the range (−57, 57) for both PEI series. All these structural variations must be reflected in the variation of chemical properties. To prove this, ten physical-chemical descriptors for C14N8&C18N10 PEIs were calculated. Among these, a significant variation of values (collected before and after docking) was shown by the Connolly accessible area and inertial principal moment of the PEIs molecules; in contrast, Wiener index and the topological diameter do not change after docking, a result just expected (these two topological descriptors follow the topology of structure and do not regard the actual geometry, thus remaining constant). Figure 4 illustrates the monitored physico-chemical properties of C14N8 PEIs series, before (in the free form) and after docking (bound) on the GOx enzyme. Each PEI structure is represented by a color. On the horizontal axis, the chemical descriptors are: 0—The origin; 1—log *P*; 2—Connolly accessible area (Å^2^); 3—Connolly molecular area (Å^2^); 4—Molecular weight; 5—Ovality; 6—Principal moment of inertia; 7—Molar refractivity (cm^3^/mol); 8—Partition coefficient, 9—Topological diameter (bonds), 10—Wiener index. On the vertical axis, the values of descriptors are represented using the same scale. Similar results, in terms of chemical descriptor variation, were obtained for the C18N10 series (see the Supplementary Material).

In order to represent in more detail the chemical property space, the above mentioned ten molecular descriptors were calculated for PEIs and joined in a 2-dimensional shape, and a surface-like radial graph was designed (Figure 5).

Each property was represented by one edge of the radial graph. LPEI 01 and BPEI 02 surfaces are not visible, being covered by BPEI 03 and BPEI 04 surfaces in the C14N8 free PEI series, while the BPEI 03 surface is not visible because those of BPEI 02 and BPEI 04 surfaces of C14N8 docked PEI series (for more details, see the Supplementary Material).

There are clear differences in the chemical property space of PEIs, before and after docking.

Comparing two molecules is equivalent to formation of a complex cluster (corresponding to their virtual interaction); according to the network theory, the complexity of a cluster formed between two PEI structures is directly proportional to their dissimilarity (*i.e.*, the more “different” the molecules are, the more complex the cluster results; see the scatterplots in Figure 2 and in the rest of the Supplementary Material).

Being located at the upper limit of PEI chemical space, BPEI 04 characterizes the upper limit of functional differentiation/functionalization, meaning in this case the highest number of functional groups and ramification (*i.e.*, branching).

The lower limit of chemical space is represented by LPEI 01 C14N8; this was taken as the reference of differentiation. Clearly, variations in the structure determine changes in the chemical property space of each PEI. Observe the ”same degree“ in changes of the property space, as suggested by the same roots of the second degree equation of BPEI 04 in the two series C14N8 and C18N10 (the root values are nearly identical; see the Supplementary Material).

**Figure 6:**
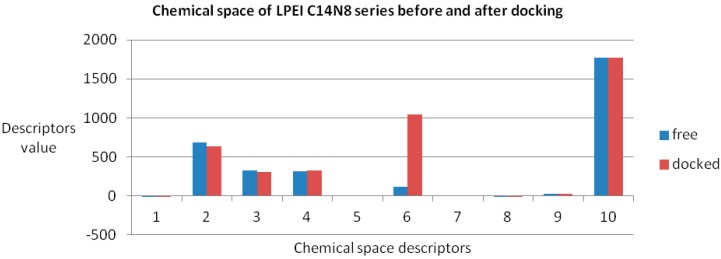
Chemical property space descriptors in C14N8 PEIs, before (free of docking) and after docking.

**Figure 7:**
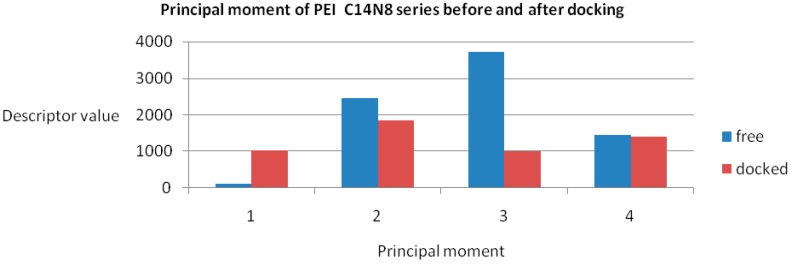
Principal moment of inertia in the C14N8 PEI series before (free of docking) and after docking.

The maximum variation was observed for BPEI 04 in both series while LPEI 01 tends to conserve its chemical properties. However, the geometry of LPEI 01 changes drastically by docking, a fact clearly evidenced by the changes in Connolly molecular surface and especially in the principal moment of inertia, computed before and after docking (see Figure 6 and Figure 7). We interpret this result as highly suggestive that LPEI 01 is more susceptible to bind at an enzyme site compared to BPEIs and meanwhile to conserve its chemical property space; it also means that its (topological/functional) structure will not be drastically affected by binding.

**Figure 8:**
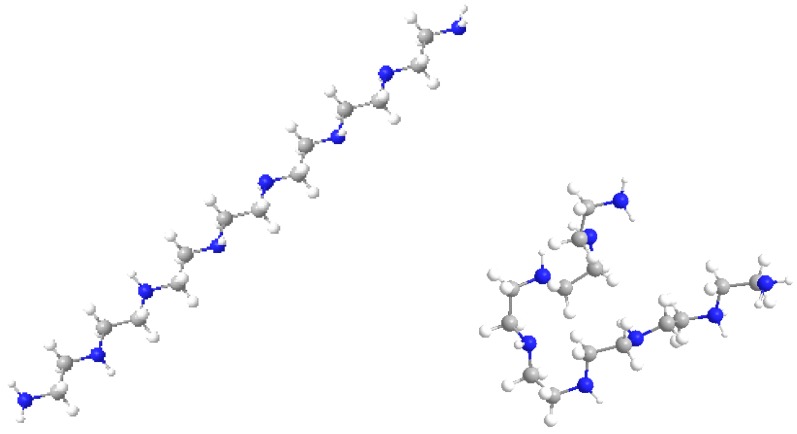
Models of LPEI 01 C14N8 before (**left**) and after docking (**right**) at GOx binding site.

Figure 8 shows the molecular models of the LPEI C14N8 structure, represented before and after docking with GOx; the changes in molecule geometry are clearly seen.

**Figure 9:**
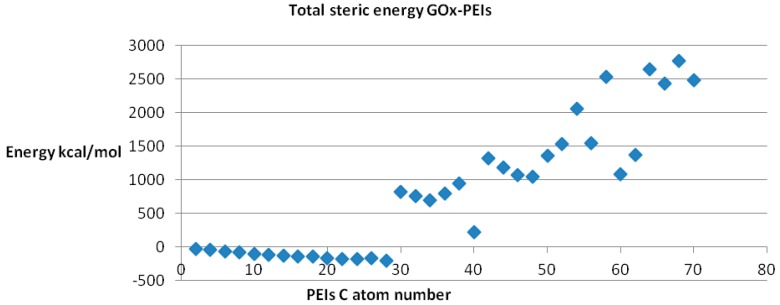
Steric energy values of GOx-LPEI complexes.

Since any biological receptor/enzyme shows saturation, (*i.e.*, a limit in the number of ligands that may be bound) in terms of its binding sites while docked being available to another ligand acting on the same binding site, we performed the docking procedure for LPEI molecules C2–C70, among which it was found feasible for C2–C28 LPEI; for the rest of LPEI molecules, the steric energy calculated for the corresponding GOx-PEI complex has positive, increased values (see Figure 9). Total hydrogen bond energy of this series (C2–C70 LPEI) varies within the range +50 and −40 kcal/mol.

Energetic data for these calculations can be found in the Supplementary Material.

**Figure 10:**
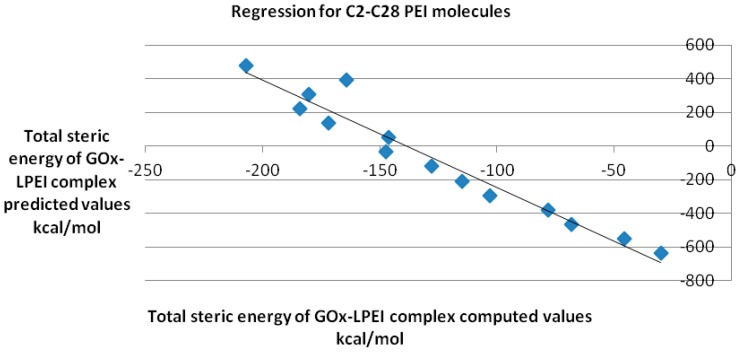
QSAR model for GOx-LPEI complex (data computed by docking).

QSAR model obtained for the reasonably docked C2–C28 molecules is represented in Figure 10; the regression equation is as follows: *y* = −6.382*x* − 884.6 with a Pearson correlation coefficient *R*^2^ of 0.907.

**Figure 11:**
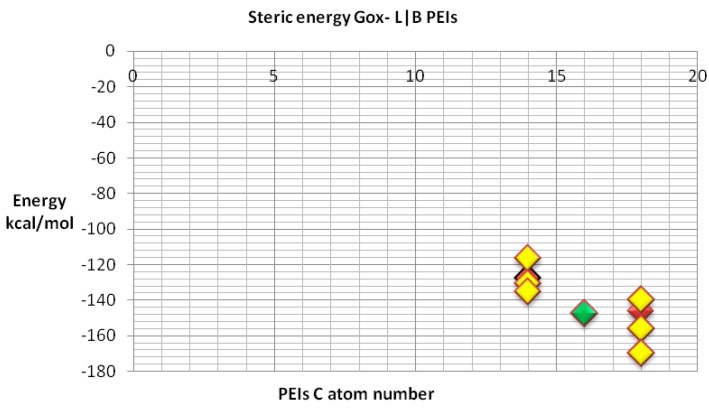
Steric energy for GOx-L/B PEI; the energy of BPEIs (C14 and C18 groups) is represented in yellow; the energy of LPEI C14 and C18 is shown in red; the energy of LPEI C16 is represented in green.

**Figure 12:**
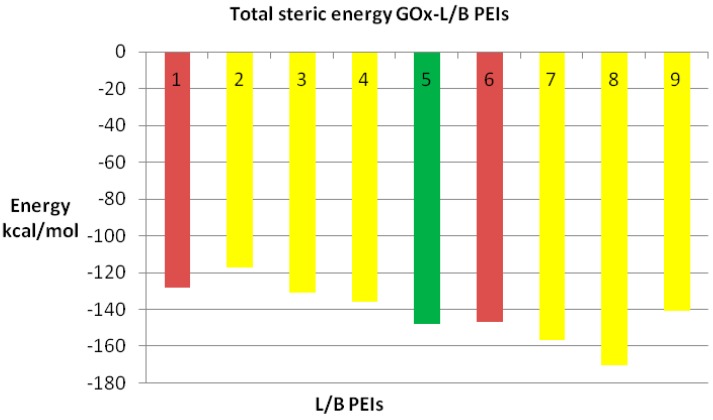
Steric energy of GOx-L/B PEIs complex (from left to right): 1 and 6 L PEI C14 and C18 (in red); 2 to 4 and 7 to 9 represent the corresponding branched isomers B PEIs (in yellow); LPEI C16 isomer is represented in green (see also Figure 11).

We also compared the docking energy (steric total energy) after docking, for LPEIs and BPEIs with similar numbers of carbon atoms, see Figure 11 and Figure 12. Nevertheless, Figure 12 gives more details on the results in Figure 11.

## 4. Conclusions

LPEIs modify their geometry more easily in comparison to BPEIs, meaning that LPEIs are more adaptable at a certain binding site. LPEIs are site-adaptive and chemical property space–stable. From the perspective of variation interval, the PEI C18N10 set is more favorable compared to C14N8 PEIs. If one wishes to build a nano-device using PEIs with GOx as a component, then LPEIs are preferred, at least in the first-generation devices (in which LPEI is docked to GOx); if additional functionalization is needed, then C18N10 PEI have to be chosen at the expense of C14N8. Also, when speaking about thermo-responsive properties, which are correlated with the principal moment of inertia, LPEIs should be chosen over BPEIs. The structural principal moment is related to temperature and thus the thermal (in)stability of the GOx-PEI complex [29]. The optimal size of PEI must be around C18N10, which is at about the middle of the length of PEI chain, as suggested by the QSAR model developed in this study, in view of getting evidence of a certain “saturation” of GOx by PEI, and *vice versa*, when the size of the PEI molecule increases. Overall, branched PEIs have a relatively small energetic influence on GOx binding when compared to linear PEIs.

## Figures and Tables

**Table 1:** Roots for the fourth-degree equations (1) and (2), Equations (3.C14) and (3.C18) in Supplementary Material.

Equation	Root 1	Root 2	Root 3	Root 4
(1)/(3.C14)	−545.181	−57.254	57.275	545.361
(2)/(3.C18)	20.376	50.003	−11.684 − 18.421*i*	−11.684 + 18.421*i*

**Table 2:** Roots for the second degree equations (3) and (4).

Equation	Root 1	Root 2
(3)	−56.9119~−57	56.9339~57
(4)	−56.917~−57	56.9586~57

## Supplementary Materials

Supplementary materials can be found at http://www.mdpi.com/1422-0067/17/4/555/s1.
