# Supplementary material for: Linear and Branched PEIs (Polyethylenimines) and Their Property Space
Source: Int J Mol Sci. 2016 Apr 13;17(4):555. doi: 10.3390/ijms17040555 (PMC4849011; doi:10.3390/ijms17040555)
Supplement: Supplementary file 1 [file ijms-17-00555-s001.pdf]

# Supplementary Material: Linear and Branched PEIs (Polyethylenimines) and Their Properties Space

**Claudiu N. Lungu, Mircea V. Diudea, Mihai V. Putz and Ireneusz P. Grudziński**

### (I) LPEI and BPEI Structures Used in Bought Groups (C14N8-C18N10)

C14N8
01

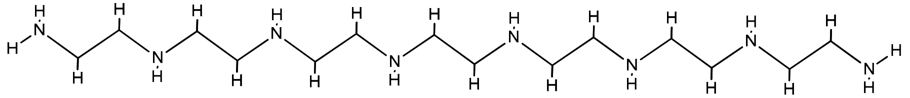

C14N8
02

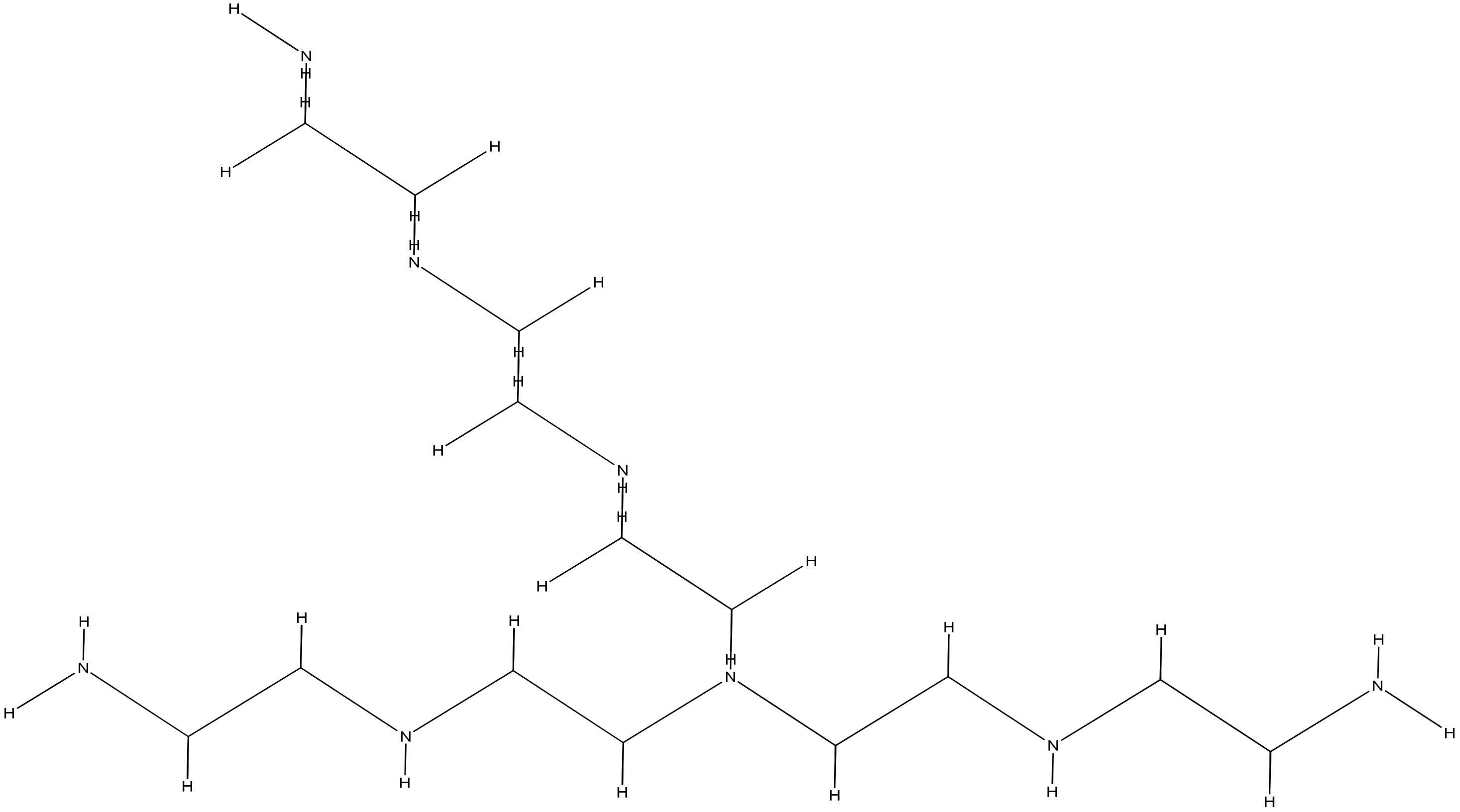

C14N8
03

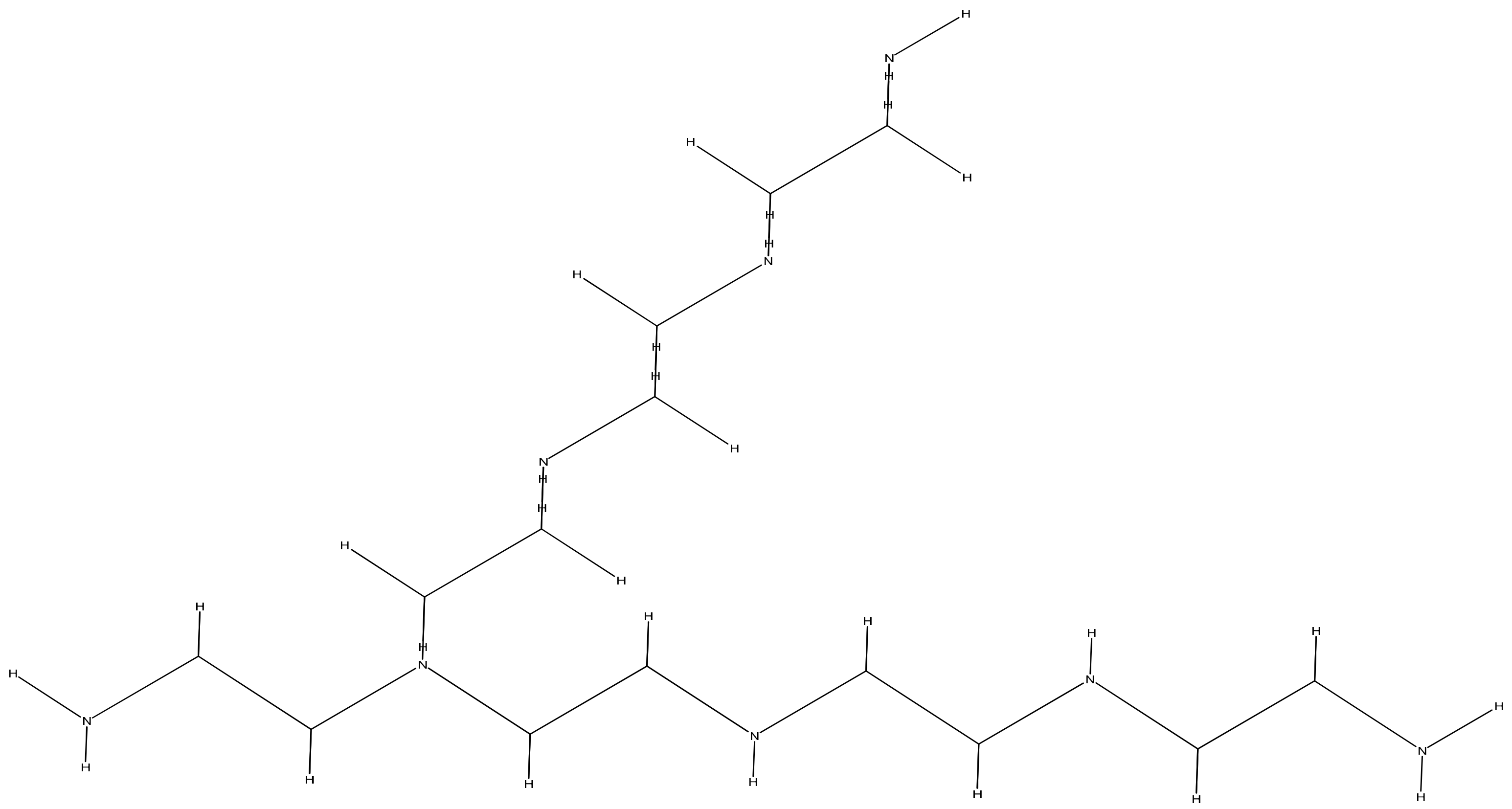

C14N8
04

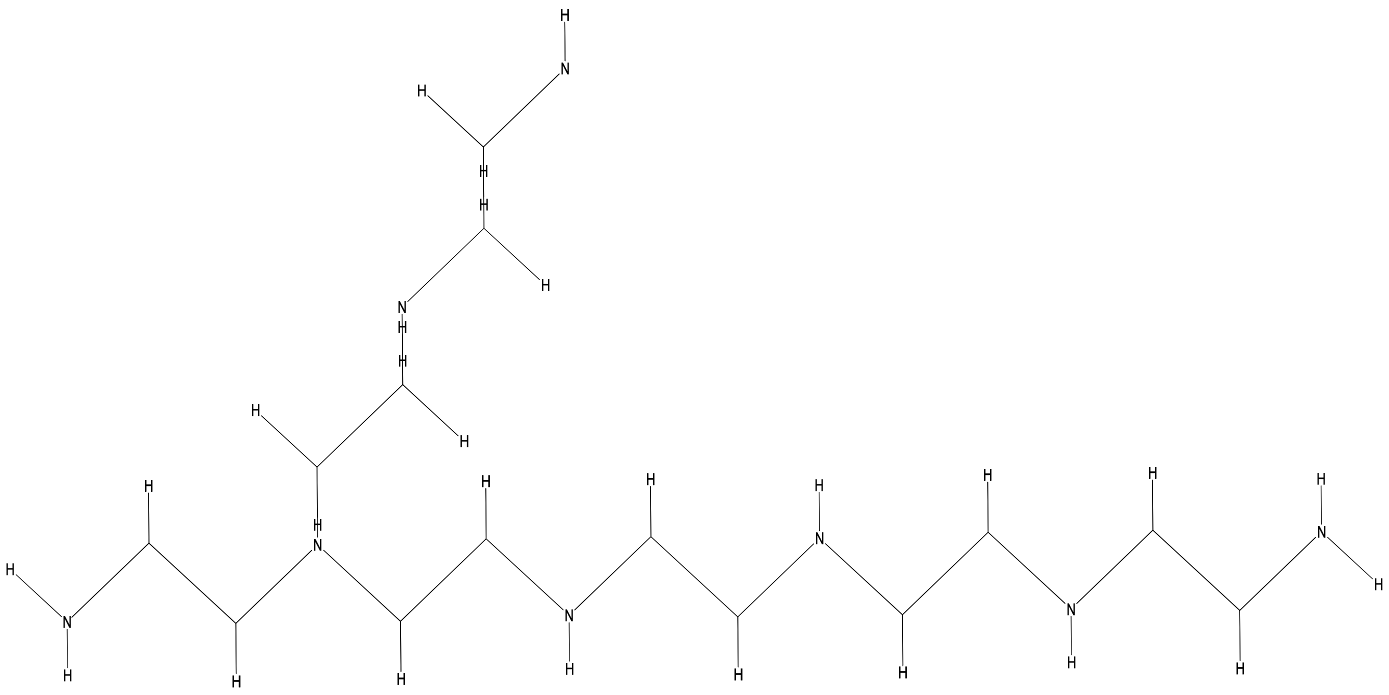

C18N10
01

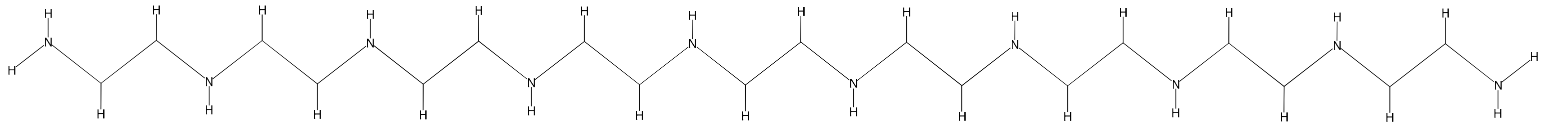

C18N10
02

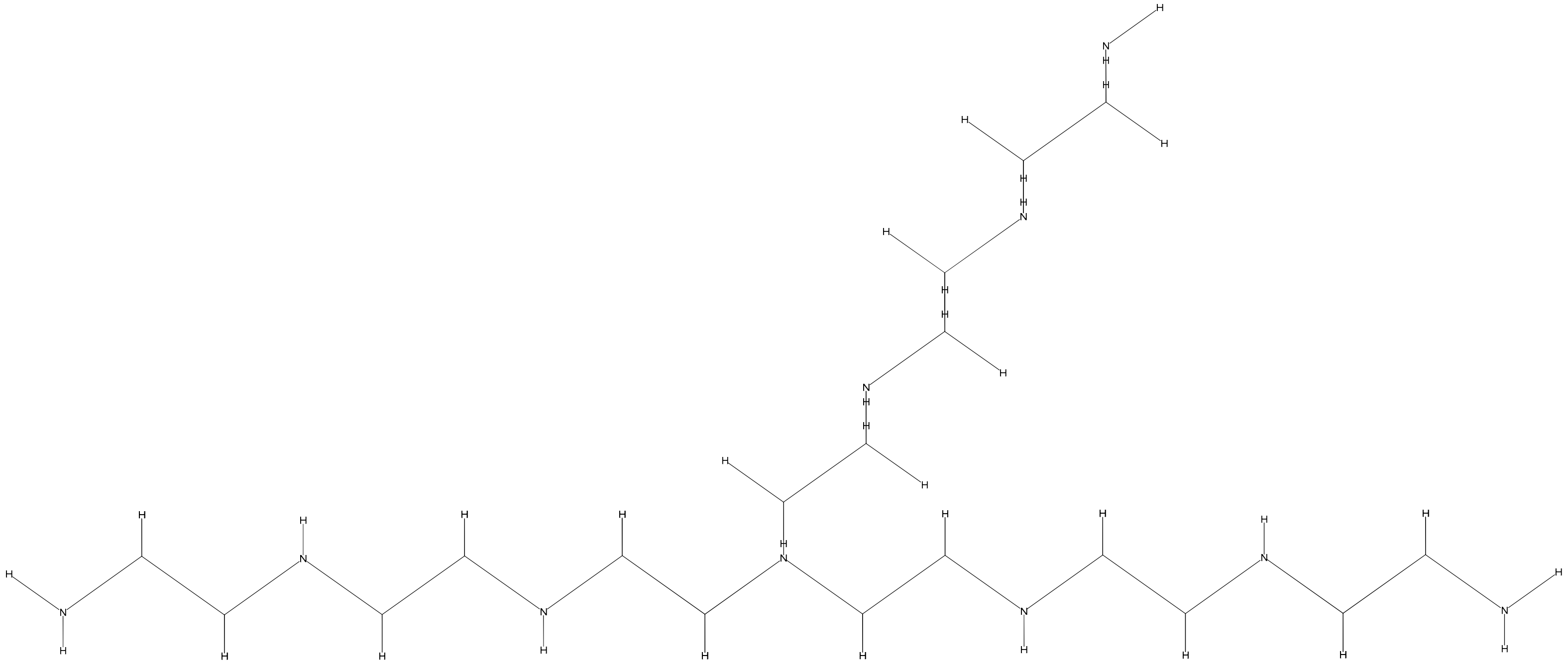

C18N10
03

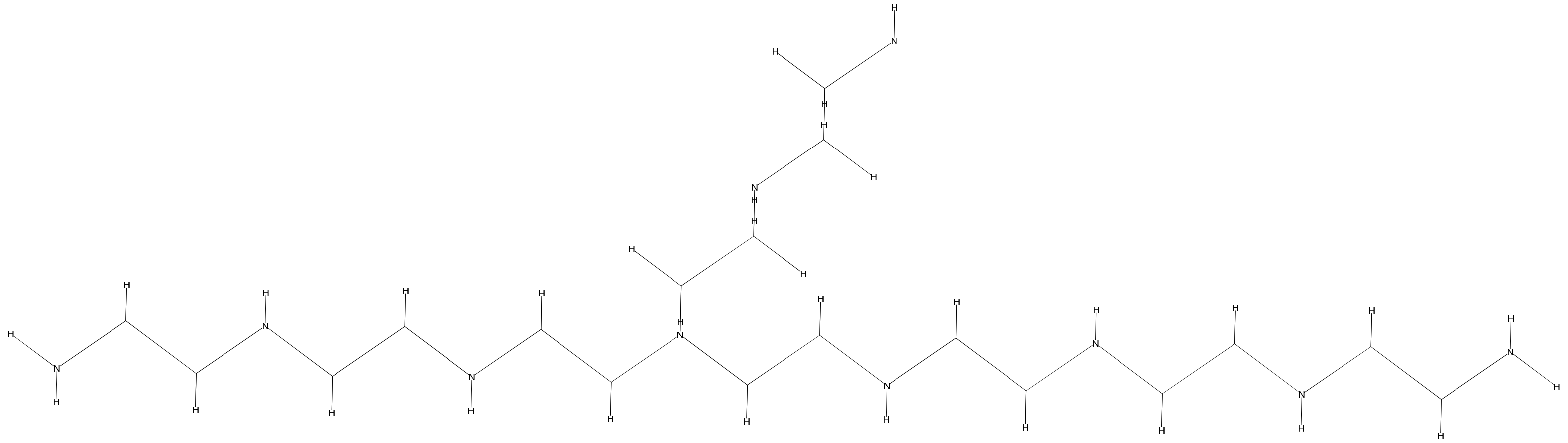

C18N10
04

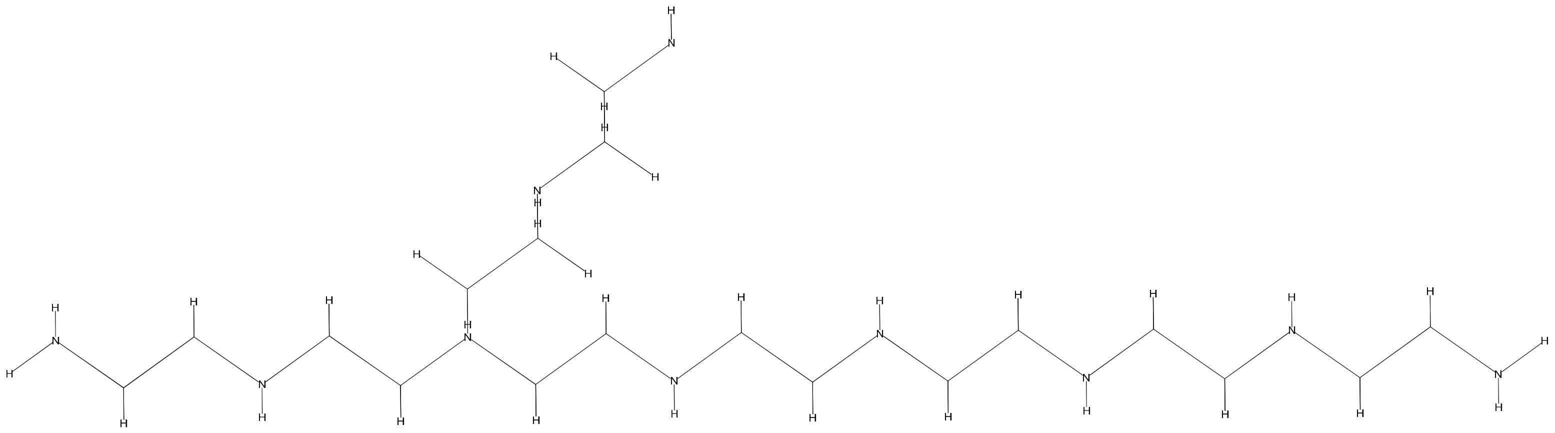

**(II) Polynomial Trend Line Cluster Equations**

**C14N8**

$y = -0.000x^4 - 1(E - 05)x^3 + 4.278x^2 + 0.675x - 13,858$ (1)
$y = -0.000x^4 - 2(E - 05)x^3 - 31.72x^2 + 0.555x + 10,279$ (2)
$y = 0.000x^4 - 2(E - 05)x^3 - 30.06x^2 + 0.661x + 97,401$ (3)
$y = +4.278x^2 + 0.675x - 13,858$ (1)
$y = -31.72x^2 + 0.555x + 10,279$ (2)
$y = -30.06x^2 + 0.661x + 97,401$ (3)

**C18N10**

$y = 0.000x^4 - 1(E - 05)x^3 - 10.91x^2 + 0.628x + 35,368$ (1)
$y = 0.000x^4 - 1(E - 05)x^3 - 21.96x^2 + 0.524x + 71,194$ (2)
$y = 0.000x^4 - 1(E - 05)x^3 - 14.96x^2 + 0.622x + 48,499$ (3)
$y = -10.91x^2 + 0.628x + 35,368$ (1)
$y = -21.96x^2 + 0.524\ x + 71,194$ (2)
$y = -14.96x^2 + 0.622x + 48,499$ (3)

**C14N8 & C18N10**

$y = 0.001x^4 - 2(E - 05)x^3 - 36.53x^2 + 0.605x + 11,837$ (7)
$y = 0.000x^4 - 2(E - 05)x^3 - 30.07x^2 + 0.661x + 97,426$ (3)
$y = 0.000x^4 - 7(E - 06)x^3 - 14.54x^2 + 0.276x + 47,143$ (6)
$y = 0.000x^4 - 2(E - 05)x^3 - 31.73x^2 + 0.555x + 10,282$ (2)
$y = 0.001x^4 - 3(E - 05)x^3 - 36.09x^2 + 0.637x + 11,694$ (5)
$y = 0.000x^4 - 2(E - 05)x^3 - 30.16x^2 + 1.061x + 97,735$ (4)
$y = -36.53x^2 + 0.605x + 11,837$ (7)
$y = -30.07x^2 + 0.661x + 97,426$ (3)
$y = -14.54x^2 + 0.276x + 47,143$ (6)
$y = -31.73x^2 + 0.555x + 10,282$ (2)
$y = -36.09x^2 + 0.637x + 11,694$ (5)
$y = -30.16x^2 + 1.061x + 97,735$ (4)

**(III) Second Degree Equations Square Roots**

**C14N8**

| Equation | Square Root 1 | Square Root 2 |
|---|---|---|
| $y = +4.278x^2 + 0.675x - 13,858$ (1) | −56.9944 | 56.8366 |
| $y = -31.72x^2 + 0.555x + 10,279$ (2) | −17.9928 | 18.0103 |
| $y = -30.06x^2 + 0.661x + 97,401$ (3) | −56.9119 | 56.9339 |

**C18N10**

| Equation | Square Root 1 | Square Root 2 |
|---|---|---|
| $y = -10.91x^2 + 0.628x + 35,368$ (1) | −56.908 | 56.9656 |
| $y = -21.96x^2 + 0.524x + 71,194$ (2) | −56.9265 | 56.9504 |
| $y = -14.96x^2 + 0.622x + 48,499$ (3) | −56.917 | 56.9586 |

## C14N8 & C18N10

| Equation | Square Root 1 | Square Root 2 |
|---|---|---|
| $y = -30.07x^2 + 0.661x + 97{,}426$ (3) | −56.9098 | 56.9318 |
| $y = -14.54x^2 + 0.276x + 47{,}143$ (6) | −56.9317 | 56.9507 |
| $y = -31.73x^2 + 0.555x + 10{,}282$ (2) | −17.9926 | 18.01 |
| $y = -36.09x^2 + 0.637x + 11{,}694$ (5) | −17.9918 | 18.0095 |
| $y = -30.16x^2 + 1.061x + 97{,}735$ (4) | −56.9082 | 56.9434 |

## (IV) Charts for Equations

## C14N8

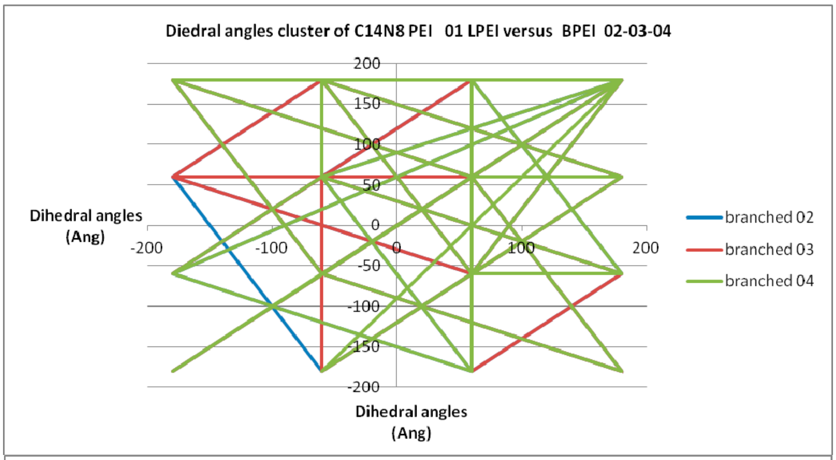


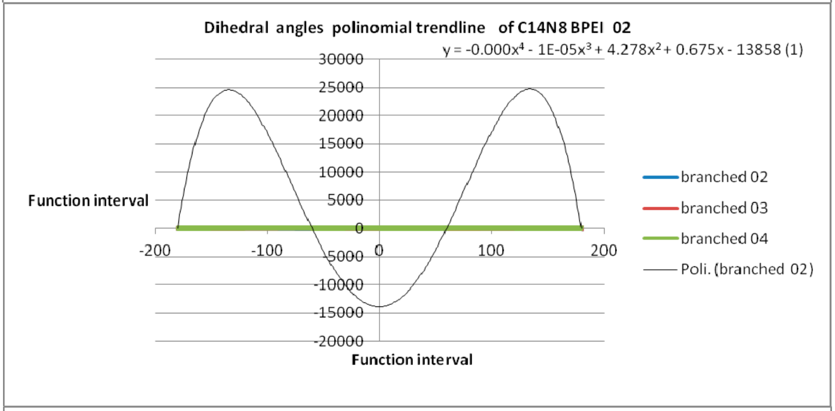


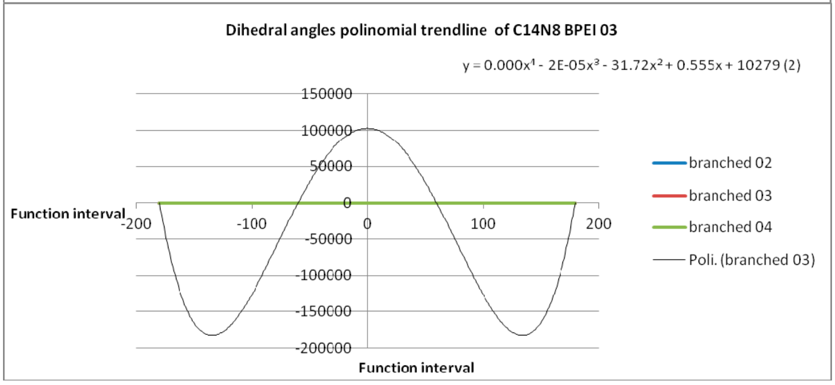

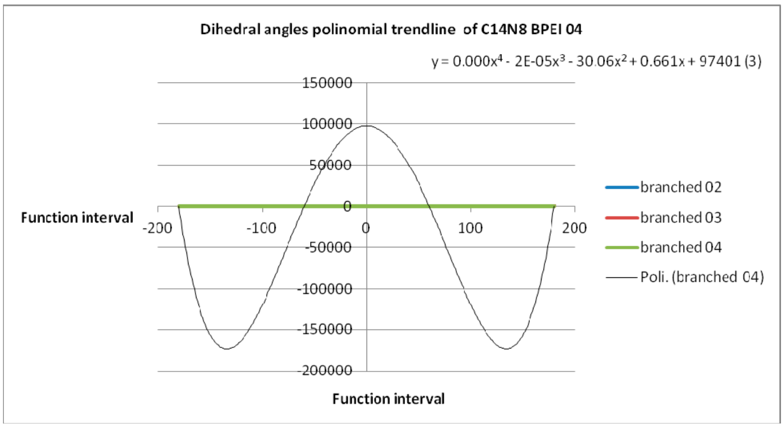
Dihedral angles polinomial trendline of C14N8 BPEI 04
y = 0.000x⁴ - 2E-05x³ - 30.06x² + 0.661x + 97401 (3)
Function interval
branched 02
branched 03
branched 04
Poli. (branched 04)
Function interval

## C18N10

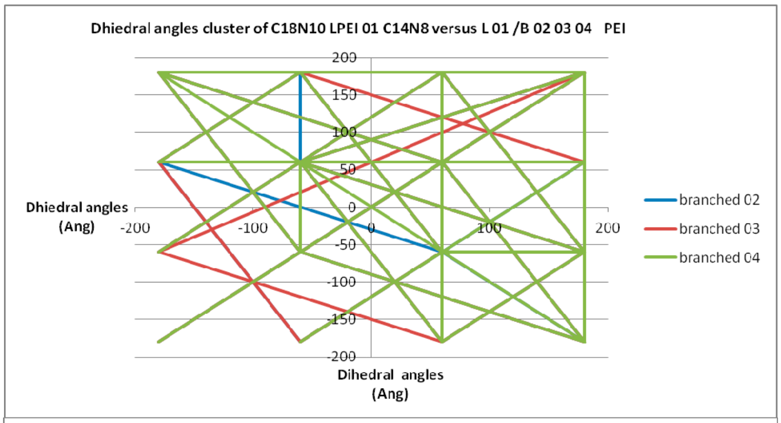
Dhiedral angles cluster of C18N10 LPEI 01 C14N8 versus L 01 /B 02 03 04 PEI
Dhiedral angles (Ang)
branched 02
branched 03
branched 04
Dihedral angles (Ang)

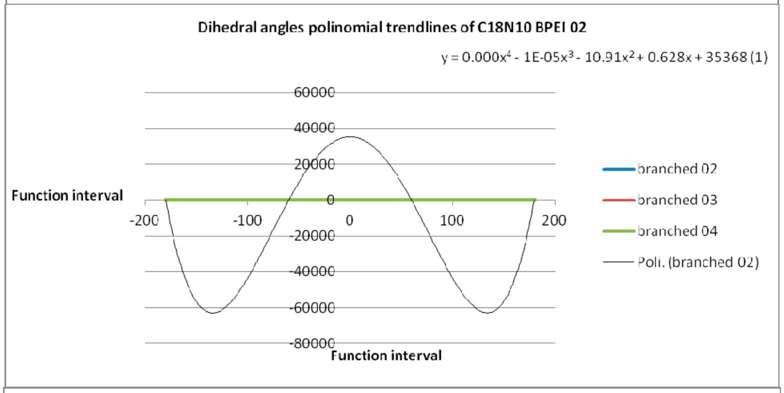
Dihedral angles polinomial trendlines of C18N10 BPEI 02
y = 0.000x⁴ - 1E-05x³ - 10.91x² + 0.628x + 35368 (1)
Function interval
branched 02
branched 03
branched 04
Poli. (branched 02)
Function interval

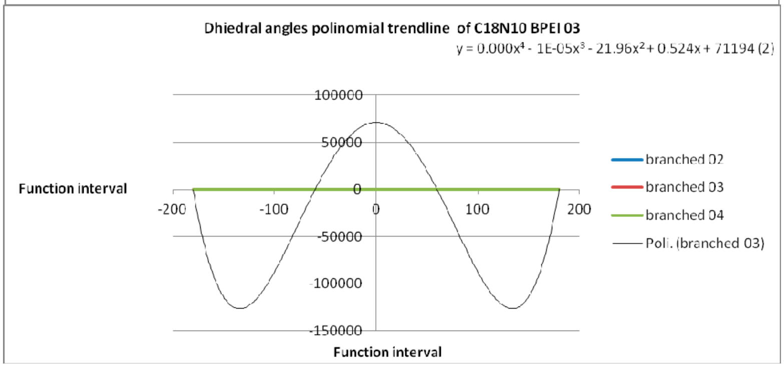
Dhiedral angles polinomial trendline of C18N10 BPEI 03
y = 0.000x⁴ - 1E-05x³ - 21.96x² + 0.524x + 71194 (2)
Function interval
branched 02
branched 03
branched 04
Poli. (branched 03)
Function interval

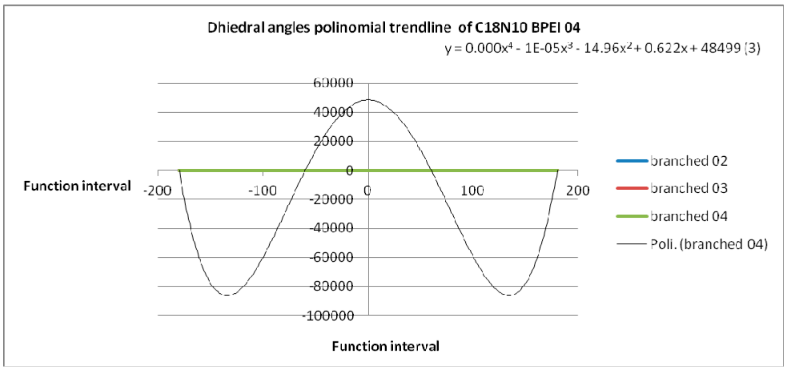


## C14N10 & C18N10

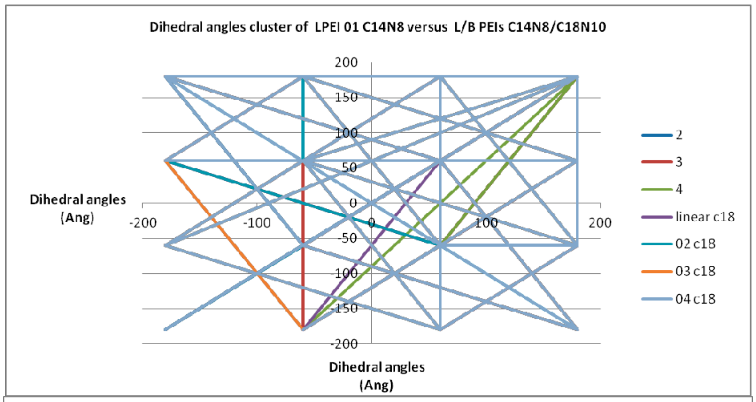


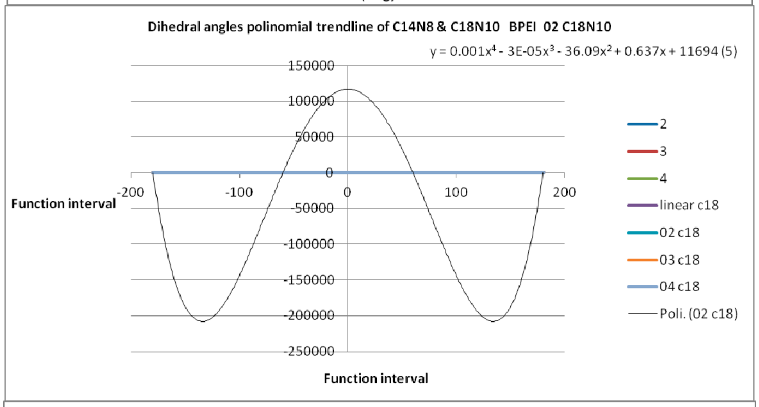


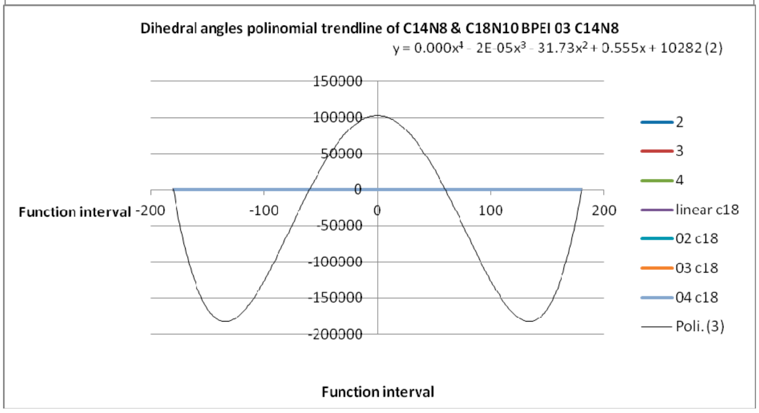

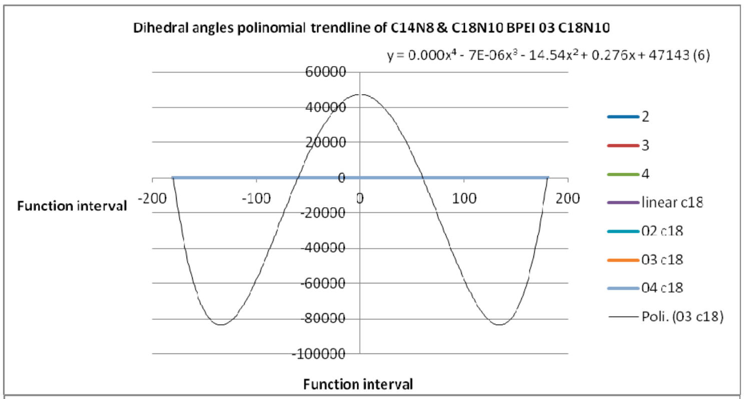
Dihedral angles polinomial trendline of C14N8 & C18N10 BPEI 03 C18N10
y = 0.000x⁴ - 7E-06x³ - 14.54x² + 0.276x + 47143 (6)
Function interval
2
3
4
linear c18
02 c18
03 c18
04 c18
Poli. (03 c18)

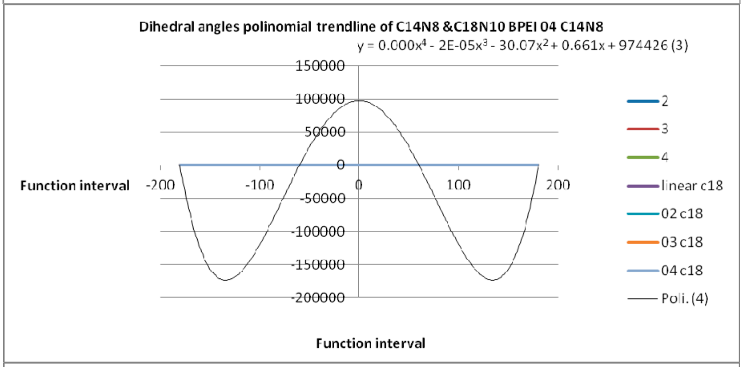
Dihedral angles polinomial trendline of C14N8 &C18N10 BPEI 04 C14N8
y = 0.000x⁴ - 2E-05x³ - 30.07x² + 0.661x + 974426 (3)
Function interval
2
3
4
linear c18
02 c18
03 c18
04 c18
Poli. (4)

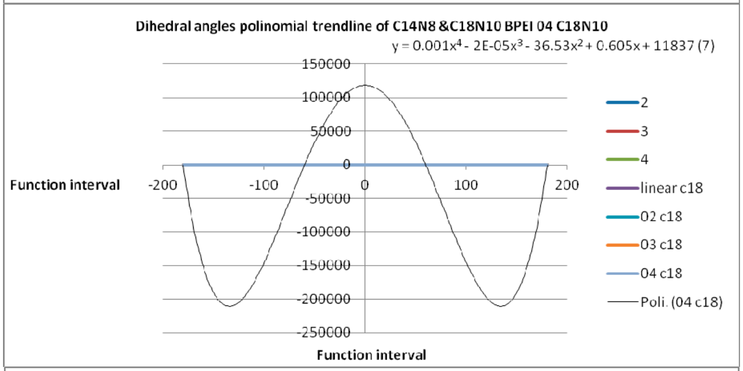
Dihedral angles polinomial trendline of C14N8 &C18N10 BPEI 04 C18N10
y = 0.001x⁴ - 2E-05x³ - 36.53x² + 0.605x + 11837 (7)
Function interval
2
3
4
linear c18
02 c18
03 c18
04 c18
Poli. (04 c18)

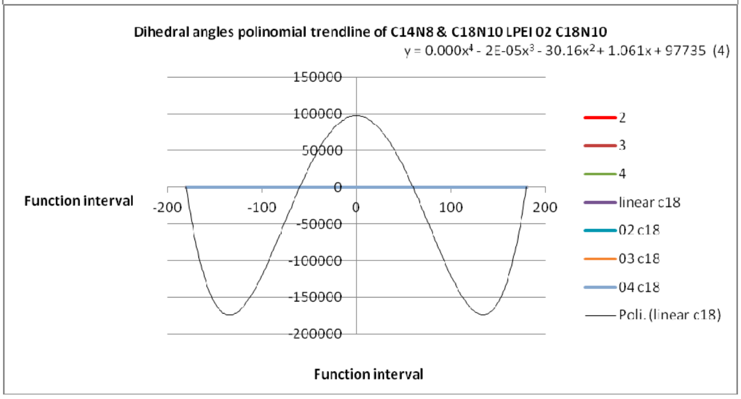
Dihedral angles polinomial trendline of C14N8 & C18N10 LPEI 02 C18N10
y = 0.000x⁴ - 2E-05x³ - 30.16x² + 1.061x + 97735 (4)
Function interval
2
3
4
linear c18
02 c18
03 c18
04 c18
Poli. (linear c18)
